# Placenta-Derived Decidua Stromal Cells: A New Frontier in the Therapy of Acute Graft-Versus-Host Disease

**DOI:** 10.1093/stmcls/sxae003

**Published:** 2024-01-10

**Authors:** Olle Ringdén, Behnam Sadeghi

**Affiliations:** Translational Cell Therapy Research (TCR), Division of Pediatrics, Department of Clinical Science, Intervention and Technology (CLINTEC), Karolinska Institutet, Huddinge, Sweden; Translational Cell Therapy Research (TCR), Division of Pediatrics, Department of Clinical Science, Intervention and Technology (CLINTEC), Karolinska Institutet, Huddinge, Sweden

**Keywords:** desidua stromal cells, acute graft-versus-host disease, allogeneic hematopoietic cell transplantation, mesenchymal stromal cells, cell therapy

## Abstract

Acute graft-versus-host disease (GVHD) is a frequent and potentially life-threatening complication following allogeneic hematopoietic cell transplantation (HCT). Mesenchymal stromal cells (MSCs), rare precursors found in all body tissues, possess immunosuppressive properties and can inhibit alloreactivity both in vitro and in vivo. Two decades ago, we introduced bone marrow-derived (BM) MSCs as a novel therapy for acute GVHD. While some patients responded to BM-MSCs, the response was not universal. Commercially available BM-MSCs are now used for acute GVHD treatment in Canada, Japan, and New Zealand. The fetus is protected from the mother’s immune system by the placenta, and our research found that placenta-derived decidua stromal cells (DSCs) offer a stronger immunosuppressive effect than other sources of stromal cells. Safety studies in rabbits, rats, mice, and humans have shown negligible or no side effects from BM-MSCs or DSCs. In a phase I/II trial for severe acute GVHD, we treated 21 patients (median age, 49 years; range 1.6-72 years) with severe biopsy-proven gastrointestinal acute GVHD. The median cell dose of DSCs was 1.2 × 10^6^ (range 0.9-2.9) cells/kg body weight, with a median of 2 (range 1-6) infusions given 1 week apart. The cell viability of DSCs was 93% (range, 69%-100%), and the median cell passage number was 4 (range, 2-4). All patients responded, with a complete response of acute GVHD in 11 patients and partial response in 10 and 1-year survival of 81%. Randomized trials are needed to prove the superiority of DSCs compared to ruxolitinib and/or other novel immunosuppressive therapies.

## Graphical Abstract

**Graphical Abstract:**
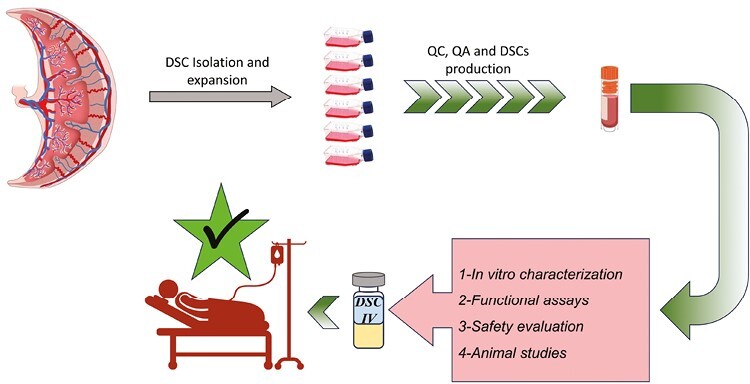

**Significance Statement**

Graft-versus-host disease (GVHD) is a serious complication after allogeneic hematopoietic cell transplantation. If patients do not respond to corticosteroids, it is fatal with slim chances of survival. Numerous treatments have been tried but often abandoned due to ineffectiveness. Mesenchyme cell therapy was introduced as a biological treatment. This article reviews existing treatments and presents the successful approach using placenta-derived mesenchymal cells (DSCs). We discussed animal studies and clinical trials using DSCs.

## Introduction

Allogeneic hematopoietic cell transplantation (HCT) is a recognized therapy for advanced hematological malignancies, primarily leukemias, aplastic anemia, and metabolic diseases.^1-3^ Graft-versus-host disease (GVHD), first described in mouse models and initially termed secondary disease,^4^ is a significant complication of HCT. Conditioning prior to HCT, which includes total body irradiation and cytotoxic drugs, causes tissue damage, leading to the release of proinflammatory cytokines. Subsequently, alloreactive donor T cells, activated by recipient and/or donor antigen-presenting cells (APCs), induce GVHD and proliferate. APCs present a variety of histocompatibility class I and class II antigens, viral antigens, or minor antigenic peptides to the donor’s alloreactive T cells. CD4+ T cells recognize antigens in association with HLA class II molecules. Cytokines stimulate CD4+ T cells, which release IL-2, activating cytotoxic CD8+ T cells that react with HLA class I positive targets.^5,6^ The primary target organs of acute GVHD are the skin, gastrointestinal tract, and liver. However, virtually all organs may be involved in GVHD, including the urinary tract, lungs, and brain.^7,8^ The pathogenesis of GVHD is also influenced by the release of danger-associated molecular patterns and tissue damage caused by granulocytes.^9,10^

However, pathogen-associated molecular patterns and other myeloid-derived cells are also important.^11,12^

The Janus kinase (JAK) and signal transducers and activators of transcription signaling pathway play a role in immune cell activation and tissue inflammation during acute GVHD.^13,14^ Tissue damage is also driven by inflammatory cytokines.

## Treatment of Acute GVHD

Over the past 2 decades, a calcineurin inhibitor combined with methotrexate has been the primary prophylaxis to prevent GVHD.^15^ More recently, post-transplant cyclophosphamide was introduced.^16,17^

For several decades, corticosteroids have been the first-line therapy for acute GVHD. Over the years, virtually all immunosuppressive therapies have been used to treat steroid-refractory acute GVHD.^18,19^ These therapies include anti-thymocyte globulin (ATG), extracorporeal photopheresis, mycophenolate mofetil, inolimomab, daclizumab, sirolimus, infliximab, alemtuzumab, methotrexate, tacrolimus, cyclophosphamide, pentostatin, etanercept, and mesenchymal stromal cells (MSCs). Patients treated for steroid-refractory acute GVHD with ATG had poor outcomes.^20^ Most secondary therapies for acute GVHD were unsuccessful.^18^ For instance, infliximab showed a complete response of 17% in one study and 62% in another, with survival rates of 17% and 38% in the 2 studies, respectively.^21^ Busca et al^22^ demonstrated that etanercept had a complete response rate of 31% in 13 patients with steroid-refractory acute GVHD. Extracorporeal photopheresis showed varying responses in different studies treating patients with grade 2-4 acute GVHD.^23,24^ Vedolizumab, an anti-α4β7 integrin antibody, showed promise in a few patients.^25^ However, vedolizumab was found to be more promising as prophylaxis against GVHD early after transplantation.^26^ In recent years, the most promising pharmacological immunosuppressive drug treatment against GVHD has been ruxolitinib, a JAK 1 and 2 inhibitor, which was studied in 2 prospective randomized trials.^27,28^ Overall responses at day 28 were 62% and 55%, significantly better than other available therapies. Overall survival at 6 months was 46% and 51% in the 2 studies, respectively. Side effects due to ruxolitinib were common and included anemia, thrombocytopenia, hypokalemia, neutropenia, edema, and infections. Based on these 2 studies, ruxolitinib is approved for the treatment of corticosteroid-refractory acute GVHD in adults and pediatric patients above 12 years of age by the US Food and Drug Administration.

## Mesenchymal Stromal Cells

Friedenstein et al^29^ were the first to describe MSCs. MSCs are very rare precursor cells (<1:10 000 cells) and are found in all tissues of the body.^30,31^ MSCs have potential in regenerative medicine and can differentiate into several cells of mesenchymal cell origin such as bone, cartilage, tendons, cardiomyocytes, and fat.^32,33^ MSCs are positive for CD29, CD73, CD90, CD105, and CD166 ^33,34^, but negative for hematopoietic markers. They express HLA class I but not HLA class II.^35^

## Immunosuppression by MSCs

MSCs inhibit T-cell alloreactivity in mixed lymphocyte reaction (MLR).^36,37^ The inhibitory effect of MSCs in MLR and suppression of T cells does not depend on HLA-compatibility between the lymphocytes and MSCs.^37^ MSCs also inhibited MLR after differentiation to bone, fat, and cartilage.^35^ MSCs increase CD4+T cells, CD25+ regulatory T cells, and IL-10 production.^38-41^ MSCs affect dendritic cells and decrease their TNF-α and IL-12 production, and promote IL-10 secretion by LPS-stimulated type 2 dendritic cells. MSCs produce several immunoregulatory factors such as HLA-G5,^42^ prostaglandin E2^38^, Galectin-1,^43^ and indoleamine-2,3-dioxygenase (IDO).^44^ IDO profoundly inhibits T cells by converting tryptophan to kynurenine.^45^ MSCs not only secrete soluble immune modulators but also have direct contact inhibitory dependent modulatory effects. This includes activation of the PD-1 pathway.^46^ MSCs also activate VCAM-1 and ICAM-1.^47^ MSCs induce Fas-mediated T-cell apoptosis.^48^ They also suppress activated T cells by upregulating CD39 and adenosine production.^49^

In mice, direct contact is important for MSCs-induced immunosuppression, and this occurs via concerted action of chemokines and nitric oxide.^50^ In mice with GVHD, it was shown that MSCs are actively induced to undergo perforin-dependent apoptosis by recipient cytotoxic T cells, a process which was essential to induce immunosuppression.^51^ Following MSCs infusion, recipient mice phagocytes engulf apoptotic MSCs and produce IDO, which was necessary to induce immune suppression. Furthermore, in nude mice, it was demonstrated that shortly after infusion, MSCs became apoptotic in the lungs and were locally eliminated by phagocytes.^52^ de Witte et al^53^ also found that MSCs-induced immunomodulation was triggered by phagocytosis of MSCs by monocytes. MSCs are rejected following infusion to major histocompatibility complex-mismatched mice.^54^ Furthermore, alloantibodies were formed after the infusion of allogeneic MSCs in baboons.^55^ Anti-HLA antibodies were not found in patients treated with MSCs for acute GVHD.^56^

Following MSCs infusions, the coagulation system is affected, and MSCs are susceptible to complement activation.^57^ After infusion, MSCs are killed by complement after contact with serum.^58^ MSCs activate coagulation factors after infusion into blood.^59^ MSCs also exhibit potent fibrinolytic properties through their expression of a diverse array of matrix metalloproteinases.^60^

## Various Sources of MSCs for Treatment of Acute GVHD

Koc et al^61^ demonstrated that it was safe to infuse various doses of MSCs into patients undergoing autologous HCT for advanced breast cancer. MSCs were also shown to prolong skin allograft survival in baboons.^36^ We found that third-party MSCs inhibited alloreactivity in mixed lymphocyte reactions.^37^ Subsequently, we treated a 9-year-old boy with devastating grade 4 acute GVHD, who dramatically responded to HLA-haploidentical maternal MSCs. Encouraged by this case, we performed a pilot study on 8 patients with steroid-refractory acute GVHD and noted a significantly better survival compared to retrospective controls treated with pharmaceutical immunosuppressive drugs.^62^ This study was followed by a European multicenter study including 55 patients with severe steroid-refractory acute GVHD.^63^ The study included 55 patients who received MSCs from various donors. Complete resolution of acute GVHD was seen in 30/55 patients. Among those patients, the 2-year survival was 52%, compared to 16% among 21 patients with partial or no response (*P* = .02). During subsequent years, there were many small studies, including a total of 190 patients, treated with BM-MSCs in doses ranging from 0.4 to 9.0 × 10^6^ cells/kg from 1 to 21 doses with a complete response of 52%, partial response 23%, and no response 25% of the patients.^64^ Subsequently, Osiris Therapeutics performed a prospective double-blind placebo-controlled phase III study, where patients with grade 2-4 GVHD were randomized to either Prochymal (remestem-L) or placebo. Overall complete response at 28 days was 45% in the Prochymal group and 46% in the placebo group.^65^ However, among 61 patients with acute GVHD of the liver, complete response was 76% in the remestem-L group and 47% in the placebo group (*P* = .03). In 71 patients with gastrointestinal acute GVHD, complete response was 88% in the remestem-L group compared to 64% in the placebo group (*P* = .02). There was also a trend for a better outcome in children than in adults. Subsequently, remestem-L was registered for the treatment of severe acute GVHD in pediatric patients in Canada and New Zealand. In Japan, the Pharmaceuticals and Medical Devices Agency approved BM-MSCs (TEMCELLR) for acute GVHD in children and adults.

In a study by Ball et al^66^ using BM-MSCs, the response for steroid-refractory acute GVHD was 65%, and 3 years survival was 57% in 37 pediatric patients. Several Brazilian centers reported responses of 50% and survival of 20% for severe acute GVHD.^67^ Platelet lysate-expanded MSCs were used to treat steroid-refractory acute GVHD in children and adults.^68^ Responses were seen in 80% of the children compared to 50% in the adults, and survival was 88% and 29% in the 2 groups, respectively (*P* = .003). A systematic review and meta-analysis to evaluate response to and survival after MSCs treatment in patients with steroid-refractory acute GVHD was performed after a search in Medline, and Cochrane databases on published studies.^69^ The study included 336 patients after the exclusion of 610 reports. Survival at 6 months after MSCs treatment was 63%. Survival did not differ with respect to age, culture medium, or dose of MSCs. A Cochrane Library analysis was performed to evaluate MSCs as treatment and prophylaxis for acute or chronic GVHD.^70^ The conclusion was that, to date, there is no evidence to support the conclusion that BM-MSCs are an effective therapy.

Bonig et al^71^ used pooled BM-MSCs, from multiple donors, and achieved an overall response of 82% and a 6-month survival of 64%. A Turkish study found that MSCs induced a complete response in 54% of the children, a partial response of 21%, and a 2-year survival of 75% among responders.^72^ MSCs were given to 58 adults with steroid-refractory acute GVHD.^73^ Response was seen in 47%, 100 days’ survival was 35%, and 2 years’ survival was 17%. Kurtzberg et al^74^ used remestemcel-L in 241 pediatric patients with steroid-refractory acute GVHD. Overall response at day +28 was 65%. Survival at 100 days was 82% among responders and 39% among nonresponders (*P* < .001). Children treated with remestemcel-L were compared to matched patients from the Mount Sinai Acute GVHD International Consortium (Magic).^75^ Children with high levels of biomarkers Reg 3a and ST2 suggesting severe GVHD were compared. The MSCs group had a 6-month survival of 64% compared to 10% for children treated with best available therapy (*P* = .01). Best available therapy included extracorporeal photopheresis, etanercept, infliximab, ruxolitinib, ATG, MMF, alemtuzumab, basiliximab, and tocilizumab.

Gregoire et al^76^ compared MSCs from different organs for treatment of GVHD in a humanized mouse model. They compared BM-, umbilical cord (UC)-, and adipose-MSCs regarding T-cell function in vitro and for treatment of acute GVHD in NOD SCID mice. The various sources of MSCs had different effects on immune cells in vitro and in vivo. However, BM-, UC-, or adipose-derived MSCs did not significantly prevent death from GVHD.

## Anti-inflammatory and Immunosuppressive Effects by Placenta-Derived Decidua Stromal Cells

The fetus is protected from the mother’s HLA haploidentical immune system by the placenta. The placenta has been successfully used to treat burn injuries in Africa for more than 100 years and has shown a potent anti-inflammatory effect.^77^ The placenta also provides a readily available source of stromal cells without any invasive procedures like BM-MSCs with no or limited ethical consideration.^31,78,79^ In vitro studies showed that stromal cells from the fetal membrane (decidua stromal cells, DSCs) had a stronger inhibition on lymphocyte proliferation in MLR assay compared to BM-MSCs and stromal cells from the cord or placenta tissue.^80^

DSCs induce FoxP3-positive regulatory T cells and inhibit alloreactivity in vivo in a contact-dependent manner as well as by soluble factors like BM-MSCs.^81^ DSCs are half the size of MSCs and do not differentiate well from chondrocytes and osteocytes.^82,83^ In MLR, DSCs promoted an anti-inflammatory cytokine profile.^81,84^ DSCs also have stronger hemostatic properties than MSCs. DSCs had a higher expression level of programmed cell death ligand-1 (PD-L1), PD-L2, and CD49d (markers for homing to inflamed tissue) than BM-MSCs.^85^ Other differences between BM-MSCs and DSCs are that the latter are more dependent on cell-to-cell contact for immunosuppression.^81^ Blocking experiments suggest that interferon-γ, prostaglandin E2, IDO, and PD-L1 are involved in the immunosuppressive mechanism of DSCs.

## Safety of BM-MSCs and Placenta-Derived DSCs

In contrast to pharmaceutical immunosuppressive agents, MSCs seem safe and there are only a few side effects reported in meta-analyses including more than 1000 patients.^86-88^ Lalu et al^87^ reported transient fever as the only side effect of MSCs treatment. In the most recent study, more than 3000 patients from 62 randomized clinical trials, treated with MSCs injections for various disorders were included.^88^ The conclusion was that MSC administration was safe in different patient populations compared with other placebo modalities.

DSCs were investigated for safety in rabbits, rats, and mice.^89,90^ In the rabbit, DSCs were infused i.a. into small arteries without adverse events.^89^ Secondly, this was administrated to Sprague-Dawley rats i.a. or i.v. without any negative effects on body weight, body temperature, activity, motility, or histological effects on internal organs. Subsequently, DSCs were given i.v. at doses ranging from 4 to 40 × 10^6^ cells/kg to 120 mice with no immediate or long-term side effects.^90^ None of the mice died from acute toxicity or adverse reactions more than 3 and 30 days after DSCs infusion. Murine blood biochemistry profiles were normal. Compared to BM-MSCs, the DSCs have better viability, are half the size, and have a stronger clotting in human blood and plasma.^83^ To conclude from these safety studies, DSCs infusions are safe with almost no side effects in doses up to 40 times higher than used clinically.

The safety studies in humans included 44 patients treated with DSCs and 40 controls.^91,92^ A median DSCs dose infused was 1.5 (range 0.9-2.9 × 10^6^ cells/kg). The patients were given a median of 2 (1-5) injections.^91^ Three patients had transient reactions during DSCs infusion. One of them had septicemia and fever and had transient chills during DSCs infusion. Another patient experienced transient vertigo. One patient experienced headache and dyspnea during the fourth DSCs infusion, reversed by oxygen support. The laboratory values, hemorrhages, causes of death, infections, and infusions were similar between the patients treated with DSCs and the controls.

To conclude from safety analysis in experimental animals and humans, DSCs seem safe even using high doses. DSCs are only half the size of BM-MSCs.^83^ To conclude from the safety studies, both BM-MSCs and DSCs seem safe and with no side effects. This is in contrast to ruxolitinib where patients often have thrombocytopenia, anemia, neutropenia, and infections.^27,28^

## DSCs for Treatment of Acute GVHD

**Figure 1.**
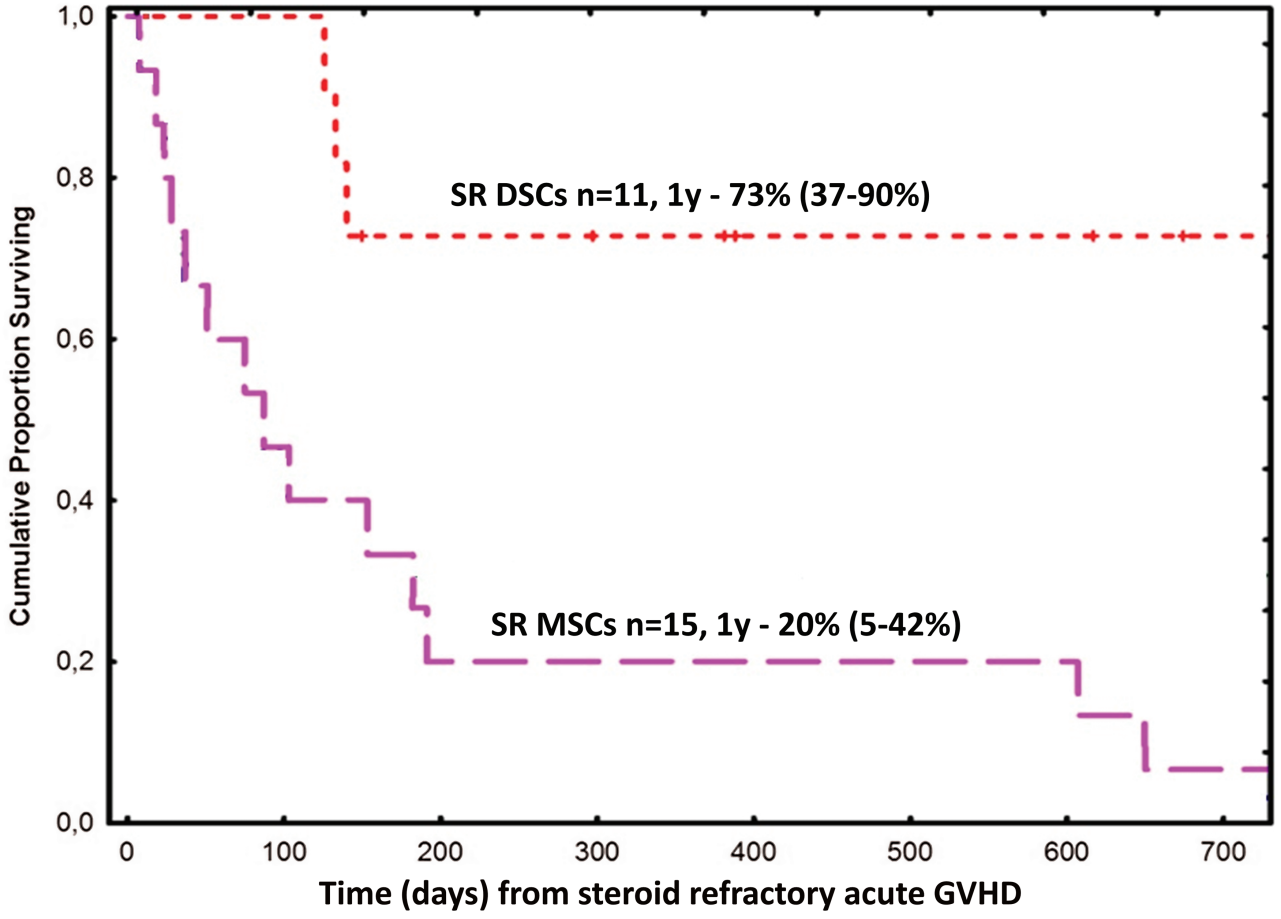
Kaplan-Meier estimate on overall survival in patients with steroid-refractory grades 3-4 acute GVHD treated with DSCs (*n* = 11) compared with retrospective controls treated with BM-MSCs (*n* = 15, *P* = .0015) at our institution, Center for Allogeneic Stem Cell Transplantation, Karolinska University Hospital.

In initial studies, DSCs were called fetal membrane cells (FMCs), because at that time we did not know that they were of maternal origin.^85^ Initially, DSCs were dissolved in AB plasma before i.v. infusion in patients with severe acute GVHD. We later found that DSCs dissolved in albumin had a significantly better viability in median 95% as compared to DSCs dissolved in AB plasma where it was 90% (*P* = .001).^92^ Patients who were treated for severe acute GVHD with DSCs dissolved in albumin had significantly better response compared to patients given DSCs dissolved in AB plasma (*P* = .01). In the former groups, 11 had complete response and 10 had partial response compared to 5 patients with partial response; 5 with complete response, and 7 with no response treated with DSCs dissolved in AB plasma. One-year survival was 76% and 47% in the 2 groups, respectively (*P* = .01). Among patients with defined steroid refractory acute GVHD, 1-year survival in patients treated with DSCs dissolved in albumin was 73%. This was significantly better than 20% in patients treated with BM-MSCs (*P* = .0015; Fig. 1).

Three patients succumbed approximately 4 months after undergoing DSC therapy. Each of these patients had achieved complete responses in their acute GVHD cases. A 42-year-old woman diagnosed with pre-B-ALL passed away due to an invasive zygomycetes infection. A 48-year-old woman with myelodysplastic syndrome who had received reduced intensity conditioning (RIC) unfortunately experienced relapse and did not survive. Lastly, a 49-year-old female diagnosed with myeloma, also treated with RIC, ultimately passed away due to recurrent disease.

**Figure 2.**
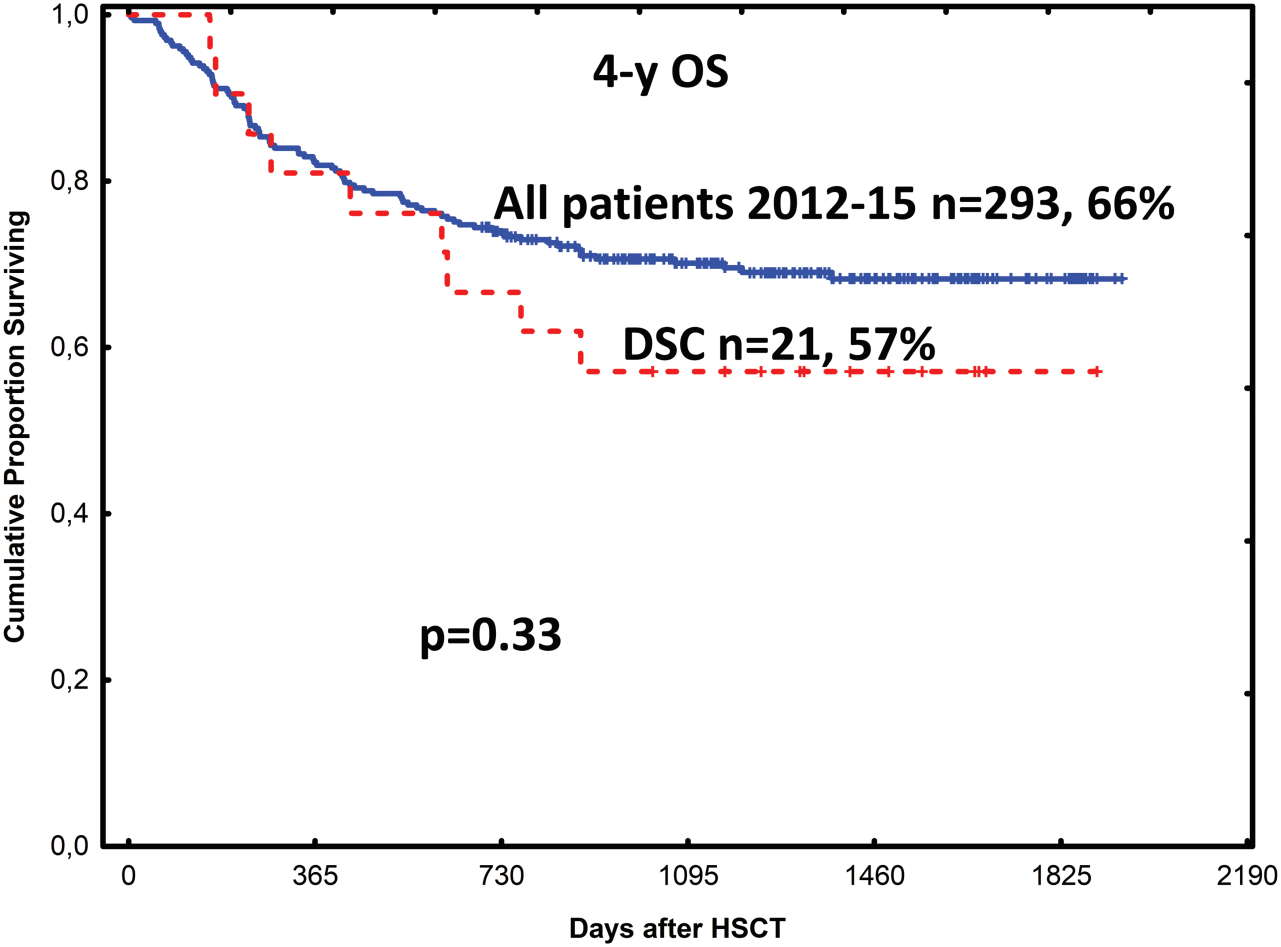
Overall survival in all patients who underwent HCT at our institution during 2012-2015 and 21 patients treated with DSCs for severe acute GVHD. Four-year survival was 66% and 57% in the 2 groups, respectively (*P* = .33).

Subsequently, we did a long-term follow-up of the 21 patients treated with DSCs dissolved in albumin.^93^ The cumulative incidence of chronic GVHD was 52%. This was mild in 6 patients, moderate in 4, and severe in 1 patient based on National Institute of Health chronic GVHD severity scoring.^94^ Nine patients died including 3 from relapse and 1 each from acute GVHD and septicemia, zygomycetes infection, liver insufficiency, cerebral hemorrhage, multiple organ failure, and chronic GVHD with obstructive bronchiolitis. At 1 year, survival was 81%. At 4 years, transplantation-related mortality was 28.6% and overall survival was 57%. Survival was similar to that for all 293 patients who underwent HCT during the same period 2012-2015 at our unit, with 66% overall survival (*P* = .33; Fig. 2).

## Discussion

There are now 20 years of experience in treating severe acute GVHD using MSCs. The by far most commonly used source of MSCs has been from BM which is readily available by aspiration from healthy donors. From meta-analysis, there is no clear-cut advantage using BM-MSCs compared to controls treated with other therapies for severe acute GVHD.^70^ Although the vast majority of patients were treated with BM-MSC, other sources such as MSCs from adipose tissue, umbilical cord, crude placenta, DSCs, and more were also evaluated.^95^ Most of the cells used did not only differ by source of stromal cells, viability, and cell dose. In addition, both human and host factors also have to be considered.^96^ Compared to all these other studies, DSCs dissolved in albumin seem to have a most promising outcome for severe acute GVHD in 21 patients with a 1-year survival of 81%.^93^

Only 3 out of the 11 patients who had steroid-refractory acute GVHD succumbed to the condition. One of these patients fell victim to an invasive fungal infection (IFI). IFIs are a prevalent occurrence post-HCT, particularly among individuals grappling with severe acute GVHD.^97^ Our observation revealed a notable incidence of IFIs in patients subjected to BM-MSC-based treatment for acute GVHD.^98^

Two patients experienced mortality due to the recurrence of their malignant diseases. This is noteworthy despite the well-documented graft-versus-leukemia effect and the anti-cancer influence associated with HCT-induced GVHD.^99-101^ Furthermore, survivors of acute GVHD display a diminished likelihood of relapse.^102^ Other recognized complications that emerge post-HCT encompass viral and bacterial infections, along with subsequent occurrences of secondary malignancies as time progresses.^103^

To comprehensively assess the potential elevation in the risk of IFIs, other infections, and disease recurrence attributed to DSCs, a substantial cohort of patients would be imperative. Similarly, the assessment of whether DSCs contribute to heightened instances of secondary malignancies would necessitate a considerable patient cohort and an extended follow-up period, extending beyond 5-10 years.

Survival also compared favorably with ruxolitinib where 6 months’ survival was around 50%.^27,28^ To be convincing for the treatment of severe acute GVHD, DSCs need to be compared to ruxolitinib or best available therapy in a prospective randomized trial. Such a prospective study requires quite a lot of financial support. This may be difficult in an academic setting due to the new laws introduced in Europe, and previously in the USA, where cell therapy is regarded as a pharmacological drug. DSCs or MSCs need to be cultured in GMP laboratories under rigorous restricted rules. The law was introduced in Europe because the big pharmaceutical companies wanted to make it very expensive with cellular therapy for small pharmaceutical companies (Åkerblom, Swedish Drug Agency, personal communication). Before the introduction of this new law, we gave thousands of cell products including HCT, BM-MSCs cells in laboratories with reversed isolation using a sterile bench, and a laminar air flow hood without having any infectious complications. The only septicemia that occurred following cell infusion was when we gave natural killer -cells prepared in our GMP laboratory.^104^ There has been no prospective trial comparing MSCs manufactured in a previously conventional lab room with a sterile bench and reversed isolation compared to an advanced and highly expensive GMP laboratory.

MSCs have only been approved for the treatment of acute GVHD in 3 countries, while their exploration is ongoing in several European countries. The restricted usage can be attributed to the outcome of a significant prospective randomized trial involving BM-MSCs, which did not achieve the primary endpoint of complete response by day 28^65^. Currently, there is insufficient convincing data to establish MSCs as a standard therapy for acute GVHD.^70^

To improve the efficacy of various MSCs products, several priming approaches to enhance efficacy were used.^105^ Priming of MSCs was attempted with cytokines and growth factors mainly with interferon-γ.^106,107^ Priming of various sources of MSCs such as umbilical cord, Wharton’s jelly, BM, placenta, adipose tissue, and cord blood were attempted by priming with hypoxia.^105^ Priming was also tried with pharmacological drugs in various disease models such as Huntington’s disease, myocardial infarction model, stroke model, excisional wounds model, emphysema model, and Ankylosing Spondylitis model. Priming of MSCs using biomaterials or different culture conditions was used in several studies and using various sources of MSCs for different diseases. Regarding DSCs as simple as changing dissolvement from plasma to albumin, significantly improved cell viability and also outcome in steroid-refractory acute GVHD.^92^ Similar improvements in culture conditionings or priming also may have improved the treatment of acute GVHD using other sources such as BM-MSCs and with MSCs from adipose, cord blood, cord, crude, placenta, or fat. DSCs from the placenta also have other advantages compared to other sources of MSCs. DSCs are readily available in large numbers because the placenta is thrown away after delivery. Furthermore, there is an unlimited supply and there is no invasive procedure. This is in contrast to bone marrow aspiration, even if this may not be very painful.

DSCs have a better expansion compared to BM-MSCs.^92^ DSCs also seem more robust than other sources of MSCs including BM-MSCs where cryopreserved cells did not well suppress T-cell proliferation in vitro. Another study confirmed that freshly cultured BM-MSCs had a stronger immunosuppressive effect than thawed frozen cells.^106,108-110^ In contrast to BM-MSCs, DSCs are more robust. Cell survival and immunosuppression by DSCs are not influenced by freeze-thawing.^111^

To improve the outcome using BM-MSCs in patients with acute GVHD, you may pool MSCs from several donors as was done by Bonig et al.^71^ Alternatively, one may select BM-MSCs, which induced cytotoxicity by the recipient immune system.^51^

MSCs affect the coagulation system and have been used to treat hemorrhages following HCT.^59,112^DSCs compared to BM-MSCs have more procoagulant tissue factor and a higher expression of CD55, complement regulatory activity.^83^Before and after infusion of MSCs and DSCs, the central venous line is flushed with low-dose heparin.^92^The safety studies have not revealed an increased risk of thrombosis following infusion of BM-MSCs or DSCs.^86,87,91,113^ In mice, 40 times higher doses of DSCs than are used clinically did not induce any thrombosis.^90^

To conclude, today, in spite of limited experience, DSCs seem so far to be the best therapy for the treatment of severe acute GVHD with no side effects and promising responses. Prospective randomized trials are needed to prove efficacy over other immunosuppressive drugs, such as ruxolitimib, and are in pipeline.

DSCs also seem superior to BM-MSCs with regard to treatment of other inflammatory disorders such as acute respiratory distress syndrome (ARDS).^114^ DSCs and possibly cord-derived MSC have shown encouraging results for the treatment of COVID-19-induced ARDS.^115^ DSCs were also successfully used in small pilot studies and anecdotal cases for hemorrhagic cystitis and polyneuropathies.^116,117^ DSCs may also be tried for many other inflammatory disorders including Crohn’s disease, ulcerative colitis, acute pancreatitis, and other inflammatory disorders due to their potent anti-inflammatory effects.

## Conclusion

DSCs are readily available following cesarean section from the fetal membrane. DSCs are easy to expand and have a stronger immunosuppressive effect in vitro and in vivo, than other sources of MSCs from the placenta or BM. Like BM-MSCs, DSCs are safe to infuse with no side effects. Using an optimal protocol, dissolving DSCs in albumin, 21 patients with severe acute GVHD, all responded with a 1-year survival of 81%. At 4 years, survival was 57%. The outcome for acute GVHD seems better than other therapies. Prospective randomized trials comparing DSCs with best available immunosuppressive therapy, like Ruxolitinib, are needed to prove efficacy. Such studies are planned.

## Data Availability

The data underlying this article will be shared on reasonable request to the corresponding author.

## References

[1] Thomas ED , Buckner CD, Banaji M, et al. One hundred patients with acute leukemia treated by chemotherapy, total body irradiation, and allogeneic marrow transplantation. Blood. 1977;49(4):511-533. 14751

[2] Storb R , Prentice RL, Thomas ED. Treatment of aplastic anemia by marrow transplantation from HLA identical siblings. Prognostic factors associated with graft versus host disease and survival. J Clin Invest. 1977;59(4):625-632. 10.1172/JCI108680 14972 PMC372266

[3] Hoogerbrugge PM , Brouwer OF, Bordigoni P, et al. Allogeneic bone marrow transplantation for lysosomal storage diseases. The European Group for Bone Marrow Transplantation. Lancet. 1995;345(8962):1398-1402. 10.1016/s0140-6736(95)92597-x 7760610

[4] Billingham RE , Brent L. A simple method for inducing tolerance of skin homografts in mice. Transplant Bull. 1957;4(2):67-71. 13422625

[5] Hill GR , Betts BC, Tkachev V, Kean LS, Blazar BR. Current concepts and advances in graft-versus-host disease immunology. Annu Rev Immunol. 2021;39:19-49. 10.1146/annurev-immunol-102119-073227 33428454 PMC8085043

[6] Ferrara JL , Smith CM, Sheets J, Reddy P, Serody JS. Altered homeostatic regulation of innate and adaptive immunity in lower gastrointestinal tract GVHD pathogenesis. J Clin Invest. 2017;127(7):2441-2451. 10.1172/JCI90592 28581444 PMC5490758

[7] Ost L , Lönnqvist B, Eriksson L, Ljungman P, Ringdén O. Hemorrhagic cystitis--a manifestation of graft versus host disease? Bone Marrow Transplant. 1987;2(1):19-25. 3139114

[8] Cooke KR , Yanik G. Acute lung injury after allogeneic stem cell transplantation: is the lung a target of acute graft-versus-host disease? Bone Marrow Transplant. 2004;34(9):753-765. 10.1038/sj.bmt.1704629 15300233

[9] Wilhelm K , Ganesan J, Müller T, et al. Graft-versus-host disease is enhanced by extracellular ATP activating P2X7R. Nat Med. 2010;16(12):1434-1438. 10.1038/nm.2242 21102458

[10] Schwab L , Goroncy L, Palaniyandi S, et al. Neutrophil granulocytes recruited upon translocation of intestinal bacteria enhance graft-versus-host disease via tissue damage. Nat Med. 2014;20(6):648-654. 10.1038/nm.3517 24836575

[11] Zitzer NC , Garzon R, Ranganathan P. Toll-like receptor stimulation by microRNAs in acute graft-vs-host disease. Front Immunol. 2018;9:2561. 10.3389/fimmu.2018.02561 30455702 PMC6230675

[12] Piper C , Drobyski WR. Inflammatory cytokine networks in gastrointestinal tract graft vs host disease. Front Immunol. 2019;10:163. 10.3389/fimmu.2019.00163 30853956 PMC6395399

[13] Stickel N , Hanke K, Marschner D, et al. MicroRNA-146a reduces MHC-II expression via targeting JAK/STAT signaling in dendritic cells after stem cell transplantation. Leukemia. 2017;31(12):2732-2741. 10.1038/leu.2017.137 28484267 PMC6231537

[14] Choi J , Cooper ML, Alahmari B, et al. Pharmacologic blockade of JAK1/JAK2 reduces GvHD and preserves the graft-versus-leukemia effect. PLoS One. 2014;9(10):e109799. 10.1371/journal.pone.0109799 25289677 PMC4188578

[15] Storb R , Deeg HJ, Fisher L, et al. Cyclosporine v methotrexate for graft-v-host disease prevention in patients given marrow grafts for leukemia: long-term follow-up of three controlled trials. Blood. 1988;71(2):293-298. 3276360

[16] Luznik L , O'Donnell PV, Symons HJ, et al. HLA-haploidentical bone marrow transplantation for hematologic malignancies using nonmyeloablative conditioning and high-dose, posttransplantation cyclophosphamide. Biol Blood Marrow Transplant. 2008;14(6):641-650. 10.1016/j.bbmt.2008.03.005 18489989 PMC2633246

[17] Saliba RM , Alousi AM, Pidala J, et al. Characteristics of graft-versus-host disease (GvHD) after post-transplantation cyclophosphamide versus conventional GvHD prophylaxis. Transplant Cell Ther. 2022;28(10):681-693. 10.1016/j.jtct.2022.07.013 35853610 PMC10141544

[18] Martin PJ , Inamoto Y, Flowers MED, Carpenter PA. Secondary treatment of acute graft-versus-host disease: a critical review. Biol Blood Marrow Transplant. 2012;18(7):982-988. 10.1016/j.bbmt.2012.04.006 22510383 PMC3386268

[19] Gatza E , Reddy P, Choi SW. Prevention and treatment of acute graft-versus-host disease in children, adolescents, and young adults. Biol Blood Marrow Transplant. 2020;26(5):e101-e112. 10.1016/j.bbmt.2020.01.004 31931115 PMC7217731

[20] Remberger M , Aschan J, Barkholt L, Tollemar J, Ringdén O. Treatment of severe acute graft-versus-host disease with anti-thymocyte globulin. Clin Transplant. 2001;15(3):147-153. 10.1034/j.1399-0012.2001.150301.x 11389703

[21] Couriel D , Saliba R, Hicks K, et al. Tumor necrosis factor-alpha blockade for the treatment of acute GVHD. Blood. 2004;104(3):649-654. 10.1182/blood-2003-12-4241 15069017

[22] Busca A , Locatelli F, Marmont F, Ceretto C, Falda M. Recombinant human soluble tumor necrosis factor receptor fusion protein as treatment for steroid refractory graft-versus-host disease following allogeneic hematopoietic stem cell transplantation. Am J Hematol. 2007;82(1):45-52. 10.1002/ajh.20752 16937391

[23] Greinix HT , Knobler RM, Worel N, et al. The effect of intensified extracorporeal photochemotherapy on long-term survival in patients with severe acute graft-versus-host disease. Haematologica. 2006;91(3):405-408. 16531267

[24] Perfetti P , Carlier P, Strada P, et al. Extracorporeal photopheresis for the treatment of steroid refractory acute GVHD. Bone Marrow Transplant. 2008;42(9):609-617. 10.1038/bmt.2008.221 18660840

[25] Forslow U , Blennow O, LeBlanc K, et al. Treatment with mesenchymal stromal cells is a risk factor for pneumonia-related death after allogeneic hematopoietic stem cell transplantation. Eur J Haematol. 2012;89(3):220-227. 10.1111/j.1600-0609.2012.01824.x 22765507

[26] Yi-Bin Chen MM , Zeiser R, Teshima T, et al. Vedolizumab for prophylaxis of lower gastrointestinal (GI) acute graft-versus-host disease (aGvHD) after allogeneic hematopoietic stem cell transplantation (allo-HSCT) from unrelated donors: results of a phase 3, randomized, double-blind, placebo-controlled, multicenter study (GRAPHITE). Paper presented at: Tandem Meetings, Transplantation & Cellular Therapy Meetings of ASTCT and CIBMTR; February 15-19, 2023; Orlando, FL. Abstract LBA2.

[27] Zeiser R , von Bubnoff N, Butler J, et al; REACH2 Trial Group. Ruxolitinib for glucocorticoid-refractory acute graft-versus-host disease. N Engl J Med. 2020;382(19):1800-1810. 10.1056/NEJMoa1917635 32320566

[28] Jagasia M , Perales MA, Schroeder MA, et al. Ruxolitinib for the treatment of steroid-refractory acute GVHD (REACH1): a multicenter, open-label phase 2 trial. Blood. 2020;135(20):1739-1749. 10.1182/blood.2020004823 32160294 PMC7229262

[29] Friedenstein AJ , Petrakova KV, Kurolesova AI, Frolova GP. Heterotopic of bone marrow. Analysis of precursor cells for osteogenic and hematopoietic tissues. Transplantation. 1968;6(2):230-247. 5654088

[30] Pittenger MF , Mackay AM, Beck SC, et al. Multilineage potential of adult human mesenchymal stem cells. Science. 1999;284(5411):143-147. 10.1126/science.284.5411.143 10102814

[31] In ‘t Anker PS , Sicco A Scherjon, Carin Kleijburg-van der Keur, et al. Isolation of mesenchymal stem cells of fetal or maternal origin from human placenta. Stem Cells. 2004;22(7):1338-1345. 15579651 10.1634/stemcells.2004-0058

[32] Caplan AI. Mesenchymal stem cells. J Orthop Res. 1991;9(5):641-650. 10.1002/jor.1100090504 1870029

[33] Toma C , Pittenger MF, Cahill KS, Byrne BJ, Kessler PD. Human mesenchymal stem cells differentiate to a cardiomyocyte phenotype in the adult murine heart. Circulation. 2002;105(1):93-98. 10.1161/hc0102.101442 11772882

[34] Prockop DJ. Marrow stromal cells as stem cells for nonhematopoietic tissues. Science. 1997;276(5309):71-74. 10.1126/science.276.5309.71 9082988

[35] Le Blanc K , Tammik C, Rosendahl K, Zetterberg E, Ringdén O. HLA expression and immunologic properties of differentiated and undifferentiated mesenchymal stem cells. Exp Hematol. 2003;31(10):890-896. 10.1016/s0301-472x(03)00110-3 14550804

[36] Bartholomew A , Sturgeon C, Siatskas M, et al. Mesenchymal stem cells suppress lymphocyte proliferation in vitro and prolong skin graft survival in vivo. Exp Hematol. 2002;30(1):42-48. 10.1016/s0301-472x(01)00769-x 11823036

[37] Le Blanc K , Tammik L, Sundberg B, Haynesworth SE, Ringdén O. Mesenchymal stem cells inhibit and stimulate mixed lymphocyte cultures and mitogenic responses independently of the major histocompatibility complex. Scand J Immunol. 2003;57(1):11-20. 10.1046/j.1365-3083.2003.01176.x 12542793

[38] Aggarwal S , Pittenger MF. Human mesenchymal stem cells modulate allogeneic immune cell responses. Blood. 2005;105(4):1815-1822. 10.1182/blood-2004-04-1559 15494428

[39] Maccario R , Podestà M, Moretta A, et al. Interaction of human mesenchymal stem cells with cells involved in alloantigen-specific immune response favors the differentiation of CD4+ T-cell subsets expressing a regulatory/suppressive phenotype. Haematologica. 2005;90(4):516-525. 15820948

[40] Rasmusson I , Ringdén O, Sundberg B, Le Blanc K. Mesenchymal stem cells inhibit lymphocyte proliferation by mitogens and alloantigens by different mechanisms. Exp Cell Res. 2005;305(1):33-41. 10.1016/j.yexcr.2004.12.013 15777785

[41] Németh K , Leelahavanichkul A, Yuen PST, et al. Bone marrow stromal cells attenuate sepsis via prostaglandin E(2)-dependent reprogramming of host macrophages to increase their interleukin-10 production. Nat Med. 2009;15(1):42-49. 10.1038/nm.1905 19098906 PMC2706487

[42] Selmani Z , Naji A, Zidi I, et al. Human leukocyte antigen-G5 secretion by human mesenchymal stem cells is required to suppress t lymphocyte and natural killer function and to induce CD4+CD25highFOXP3+ regulatory T cells. Stem Cells. 2008;26(1):212-222. 10.1634/stemcells.2007-0554 17932417

[43] Gieseke F , Böhringer J, Bussolari R, et al. Human multipotent mesenchymal stromal cells use galectin-1 to inhibit immune effector cells. Blood. 2010;116(19):3770-3779. 10.1182/blood-2010-02-270777 20644118

[44] Meisel R , Zibert A, Laryea M, et al. Human bone marrow stromal cells inhibit allogeneic T-cell responses by indoleamine 2,3-dioxygenase-mediated tryptophan degradation. Blood. 2004;103(12):4619-4621. 10.1182/blood-2003-11-3909 15001472

[45] Munn DH , Zhou M, Attwood JT, et al. Prevention of allogeneic fetal rejection by tryptophan catabolism. Science. 1998;281(5380):1191-1193. 10.1126/science.281.5380.1191 9712583

[46] Augello A , Tasso R, Negrini SM, et al. Bone marrow mesenchymal progenitor cells inhibit lymphocyte proliferation by activation of the programmed death 1 pathway. Eur J Immunol. 2005;35(5):1482-1490. 10.1002/eji.200425405 15827960

[47] Ren G , Zhao X, Zhang L, et al. Inflammatory cytokine-induced intercellular adhesion molecule-1 and vascular cell adhesion molecule-1 in mesenchymal stem cells are critical for immunosuppression. J Immunol. 2010;184(5):2321-2328. 10.4049/jimmunol.0902023 20130212 PMC2881946

[48] Akiyama K , Chen C, Wang DD, et al. Mesenchymal-stem-cell-induced immunoregulation involves FAS-ligand-/FAS-mediated T cell apoptosis. Cell Stem Cell. 2012;10(5):544-555. 10.1016/j.stem.2012.03.007 22542159 PMC3348385

[49] Saldanha-Araujo F , Ferreira FIS, Palma PV, et al. Mesenchymal stromal cells up-regulate CD39 and increase adenosine production to suppress activated T-lymphocytes. Stem Cell Res. 2011;7(1):66-74. 10.1016/j.scr.2011.04.001 21546330

[50] Ren G , Zhang L, Zhao X, et al. Mesenchymal stem cell-mediated immunosuppression occurs via concerted action of chemokines and nitric oxide. Cell Stem Cell. 2008;2(2):141-150. 10.1016/j.stem.2007.11.014 18371435

[51] Galleu A , Riffo-Vasquez Y, Trento C, et al. Apoptosis in mesenchymal stromal cells induces in vivo recipient-mediated immunomodulation. Sci Transl Med. 2017;9(416):eaam7828. 10.1126/scitranslmed.aam7828 29141887

[52] Pang SHM , D'Rozario J, Mendonca S, et al. Mesenchymal stromal cell apoptosis is required for their therapeutic function. Nat Commun. 2021;12(1):6495. 10.1038/s41467-021-26834-3 34764248 PMC8586224

[53] de Witte SFH , Luk F, Sierra Parraga JM, et al. Immunomodulation by therapeutic mesenchymal stromal cells (MSC) is triggered through phagocytosis of MSC by monocytic cells. Stem Cells. 2018;36(4):602-615. 10.1002/stem.2779 29341339

[54] Eliopoulos N , Stagg J, Lejeune L, Pommey S, Galipeau J. Allogeneic marrow stromal cells are immune rejected by MHC class I- and class II-mismatched recipient mice. Blood. 2005;106(13):4057-4065. 10.1182/blood-2005-03-1004 16118325

[55] Beggs KJ , Lyubimov A, Borneman JN, et al. Immunologic consequences of multiple, high-dose administration of allogeneic mesenchymal stem cells to baboons. Cell Transplant. 2006;15(8-9):711-721. 10.3727/000000006783981503 17269442

[56] Sundin M , Ringdén O, Sundberg B, et al. No alloantibodies against mesenchymal stromal cells, but presence of anti-fetal calf serum antibodies, after transplantation in allogeneic hematopoietic stem cell recipients. Haematologica. 2007;92(9):1208-1215. 10.3324/haematol.11446 17666368

[57] Moll G , Jitschin R, von Bahr L, et al. Mesenchymal stromal cells engage complement and complement receptor bearing innate effector cells to modulate immune responses. PLoS One. 2011;6(7):e21703. 10.1371/journal.pone.0021703 21747949 PMC3128611

[58] Li Y , Lin F. Mesenchymal stem cells are injured by complement after their contact with serum. Blood. 2012;120(17):3436-3443. 10.1182/blood-2012-03-420612 22966167 PMC3482856

[59] Moll G , Rasmusson-Duprez I, von Bahr L, et al. Are therapeutic human mesenchymal stromal cells compatible with human blood? Stem Cells. 2012;30(7):1565-1574. 10.1002/stem.1111 22522999

[60] Moll G , Ankrum JA, Kamhieh-Milz J, et al. Intravascular mesenchymal stromal/stem cell therapy product diversification: time for new clinical guidelines. Trends Mol Med. 2019;25(2):149-163. 10.1016/j.molmed.2018.12.006 30711482

[61] Koç ON , Gerson SL, Cooper BW, et al. Rapid hematopoietic recovery after coinfusion of autologous-blood stem cells and culture-expanded marrow mesenchymal stem cells in advanced breast cancer patients receiving high-dose chemotherapy. J Clin Oncol. 2000;18(2):307-316. 10.1200/JCO.2000.18.2.307 10637244

[62] Ringden O , Uzunel M, Rasmusson I, et al. Mesenchymal stem cells for treatment of therapy-resistant graft-versus-host disease. Transplantation. 2006;81(10):1390-1397. 10.1097/01.tp.0000214462.63943.14 16732175

[63] Le Blanc K , Frassoni F, Ball L, et al; Developmental Committee of the European Group for Blood and Marrow Transplantation. Mesenchymal stem cells for treatment of steroid-resistant, severe, acute graft-versus-host disease: a phase II study. Lancet. 2008;371(9624):1579-1586. 10.1016/S0140-6736(08)60690-X 18468541

[64] Ringdén O. Mesenchymal Stem Cell Therapy. 2013, USA: Springer.

[65] Kebriaei P , Hayes J, Daly A, et al. A Phase 3 randomized study of remestemcel-L versus placebo added to second-line therapy in patients with steroid-refractory acute graft-versus-host disease. Biol Blood Marrow Transplant. 2020;26(5):835-844. 10.1016/j.bbmt.2019.08.029 31505228 PMC7060124

[66] Ball LM , Bernardo ME, Roelofs H, et al. Multiple infusions of mesenchymal stromal cells induce sustained remission in children with steroid-refractory, grade III-IV acute graft-versus-host disease. Br J Haematol. 2013;163(4):501-509. 10.1111/bjh.12545 23992039

[67] Dotoli GM , De Santis GC, Orellana MD, et al. Mesenchymal stromal cell infusion to treat steroid-refractory acute GvHD III/IV after hematopoietic stem cell transplantation. Bone Marrow Transplant. 2017;52(6):859-862. 10.1038/bmt.2017.35 28287644

[68] Salmenniemi U , Itälä-Remes M, Nystedt J, et al. Good responses but high TRM in adult patients after MSC therapy for GvHD. Bone Marrow Transplant. 2017;52(4):606-608. 10.1038/bmt.2016.317 27941780

[69] Hashmi S , Ahmed M, Murad MH, et al. Survival after mesenchymal stromal cell therapy in steroid-refractory acute graft-versus-host disease: systematic review and meta-analysis. Lancet Haematol. 2016;3(1):e45-e52. 10.1016/S2352-3026(15)00224-0 26765648

[70] Fisher SA , Cutler A, Doree C, et al. Mesenchymal stromal cells as treatment or prophylaxis for acute or chronic graft-versus-host disease in haematopoietic stem cell transplant (HSCT) recipients with a haematological condition. Cochrane Database Syst Rev. 2019;1(1):CD009768. 10.1002/14651858.CD009768.pub2 30697701 PMC6353308

[71] Bonig H , Kuçi Z, Kuçi S, et al. Children and adults with refractory acute graft-versus-host disease respond to treatment with the mesenchymal stromal cell preparation “MSC-FFM”-outcome report of 92 patients. Cells. 2019;8(12):1577. 10.3390/cells8121577 31817480 PMC6952775

[72] Erbey F , Atay D, Akcay A, Ovali E, Ozturk G. Mesenchymal stem cell treatment for steroid refractory graft-versus-host disease in children: a pilot and first study from Turkey. Stem Cells Int. 2016;2016:1641402. 10.1155/2016/1641402 26783400 PMC4691494

[73] von Dalowski F , Kramer M, Wermke M, et al. Mesenchymal stromal cells for treatment of acute steroid-refractory graft versus host disease: clinical responses and long-term outcome. Stem Cells. 2016;34(2):357-366. 10.1002/stem.2224 26418955

[74] Kurtzberg J , Prockop S, Chaudhury S, et al; MSB-275 Study Group. Study 275: updated expanded access program for remestemcel-l in steroid-refractory acute graft-versus-host disease in children. Biol Blood Marrow Transplant. 2020;26(5):855-864. 10.1016/j.bbmt.2020.01.026 32044400 PMC8292970

[75] Kasikis S , Baez J, Gandhi I, et al. Mesenchymal stromal cell therapy induces high responses and survival in children with steroid refractory GVHD and poor risk biomarkers. Bone Marrow Transplant. 2021;56(11):2869-2870. 10.1038/s41409-021-01442-3 34471240 PMC9840529

[76] Grégoire C , Ritacco C, Hannon M, et al. Comparison of mesenchymal stromal cells from different origins for the treatment of graft-vs-host-disease in a humanized mouse model. Front Immunol. 2019;10:619. 10.3389/fimmu.2019.00619 31001253 PMC6454068

[77] Kesting MR , Wolff KD, Hohlweg-Majert B, Steinstraesser L. The role of allogenic amniotic membrane in burn treatment. J Burn Care Res. 2008;29(6):907-916. 10.1097/BCR.0b013e31818b9e40 18849850

[78] Brooke G , Rossetti T, Pelekanos R, et al. Manufacturing of human placenta-derived mesenchymal stem cells for clinical trials. Br J Haematol. 2009;144(4):571-579. 10.1111/j.1365-2141.2008.07492.x 19077161

[79] Parolini O , Alviano F, Bagnara GP, et al. Concise review: isolation and characterization of cells from human term placenta: outcome of the first international Workshop on Placenta Derived Stem Cells. Stem Cells. 2008;26(2):300-311. 10.1634/stemcells.2007-0594 17975221

[80] Karlsson H , Erkers T, Nava S, et al. Stromal cells from term fetal membrane are highly suppressive in allogeneic settings in vitro. Clin Exp Immunol. 2012;167(3):543-555. 10.1111/j.1365-2249.2011.04540.x 22288598 PMC3374287

[81] Erkers T , Nava S, Yosef J, Ringdén O, Kaipe H. Decidual stromal cells promote regulatory T cells and suppress alloreactivity in a cell contact-dependent manner. Stem Cells Dev. 2013;22(19):2596-2605. 10.1089/scd.2013.0079 23701127

[82] Erkers T , Kaipe H, Nava S, et al. Treatment of severe chronic graft-versus-host disease with decidual stromal cells and tracing with (111)indium radiolabeling. Stem Cells Dev. 2015;24(2):253-263. 10.1089/scd.2014.0265 25162829 PMC4291217

[83] Moll G , Ignatowicz L, Catar R, et al. Different procoagulant activity of therapeutic mesenchymal stromal cells derived from bone marrow and placental decidua. Stem Cells Dev. 2015;24(19):2269-2279. 10.1089/scd.2015.0120 26192403

[84] Ferrara JL , Levine JE, Reddy P, Holler E. Graft-versus-host disease. Lancet. 2009;373(9674):1550-1561. 10.1016/S0140-6736(09)60237-3 19282026 PMC2735047

[85] Ringdén O , Erkers T, Nava S, et al. Fetal membrane cells for treatment of steroid-refractory acute graft-versus-host disease. Stem Cells. 2013;31(3):592-601. 10.1002/stem.1314 23307526

[86] Thompson M , Mei SHJ, Wolfe D, et al. Cell therapy with intravascular administration of mesenchymal stromal cells continues to appear safe: an updated systematic review and meta-analysis. EClinicalMedicine. 2020;19:100249. 10.1016/j.eclinm.2019.100249 31989101 PMC6970160

[87] Lalu MM , McIntyre L, Pugliese C, et al; Canadian Critical Care Trials Group. Safety of cell therapy with mesenchymal stromal cells (SafeCell): a systematic review and meta-analysis of clinical trials. PLoS One. 2012;7(10):e47559. 10.1371/journal.pone.0047559 23133515 PMC3485008

[88] Wang Y , Yi H, Song Y. The safety of MSC therapy over the past 15 years: a meta-analysis. Stem Cell Res Ther. 2021;12(1):545. 10.1186/s13287-021-02609-x 34663461 PMC8522073

[89] Arnberg F , Lundberg J, Olsson A, et al. Intra-arterial administration of placenta-derived decidual stromal cells to the superior mesenteric artery in the rabbit: distribution of cells, feasibility, and safety. Cell Transplant. 2016;25(2):401-410. 10.3727/096368915X688191 25976072

[90] Sadeghi B , Moretti G, Arnberg F, et al. Preclinical toxicity evaluation of clinical grade placenta-derived decidua stromal cells. Front Immunol. 2019;10:2685. 10.3389/fimmu.2019.02685 31803191 PMC6877599

[91] Baygan A , Aronsson-Kurttila W, Moretti G, et al. Safety and side effects of using placenta-derived decidual stromal cells for graft-versus-host disease and hemorrhagic cystitis. Front Immunol. 2017;8:795. 10.3389/fimmu.2017.00795 28744284 PMC5504152

[92] Ringden O , Baygan A, Remberger M, et al. Placenta-derived decidua stromal cells for treatment of severe acute graft-versus-host disease. Stem Cells Transl Med. 2018;7(4):325-331. 10.1002/sctm.17-0167 29533533 PMC5866941

[93] Sadeghi B , Mats Remberger, Britt Gustafsson, et al. Long-term follow-up of a pilot study using placenta-derived decidua stromal cells for severe acute graft-versus-host disease. Biol Blood Marrow Transplant. 2019;25(10):1965-1969. 10.1016/j.bbmt.2019.05.03 31173898

[94] Filipovich AH , Weisdorf D, Pavletic S, et al. National Institutes of Health consensus development project on criteria for clinical trials in chronic graft-versus-host disease: I. Diagnosis and staging working group report. Biol Blood Marrow Transplant. 2005;11(12):945-956. 10.1016/j.bbmt.2005.09.004 16338616

[95] Trento C , Bernardo ME, Nagler A, et al. Manufacturing mesenchymal stromal cells for the treatment of graft-versus-host disease: a survey among centers affiliated with the European society for blood and marrow transplantation. Biol Blood Marrow Transplant. 2018;24(11):2365-2370. 10.1016/j.bbmt.2018.07.015 30031938 PMC6299357

[96] Phinney DG , Galipeau J, Krampera M, et al. MSCs: science and trials. Nat Med. 2013;19(7):812. 10.1038/nm.3220 23836216

[97] Tollemar J , Ringdén O, Boström L, Nilsson B, Sundberg B. Variables predicting deep fungal infections in bone marrow transplant recipients. Bone Marrow Transplant. 1989;4(6):635-641. 2555004

[98] Remberger M , Ringden O. Treatment of severe acute graft-versus-host disease with mesenchymal stromal cells: a comparison with non-MSC treated patients. Int J Hematol. 2012;96(6):822-824. 10.1007/s12185-012-1218-3 23143687

[99] Weiden PL , Flournoy N, Thomas ED, et al. Antileukemic effect of graft-versus-host disease in human recipients of allogeneic-marrow grafts. N Engl J Med. 1979;300(19):1068-1073. 10.1056/NEJM197905103001902 34792

[100] Horowitz MM , Gale RP, Sondel PM, et al. Graft-versus-leukemia reactions after bone marrow transplantation. Blood. 1990;75(3):555-562. 2297567

[101] Ringden O , Karlsson H, Olsson R, Omazic B, Uhlin M. The allogeneic graft-versus-cancer effect. Br J Haematol. 2009;147(5):614-633. 10.1111/j.1365-2141.2009.07886.x 19735262

[102] Ringden O , Labopin M, Sadeghi B, et al. What is the outcome in patients with acute leukaemia who survive severe acute graft-versus-host disease? J Intern Med. 2018;283(2):166-177. 10.1111/joim.12695 29027756

[103] Curtis RE , Rowlings PA, Deeg HJ, et al. Solid cancers after bone marrow transplantation. N Engl J Med. 1997;336(13):897-904. 10.1056/NEJM199703273361301 9070469

[104] Barkholt L , Alici E, Conrad R, et al. Safety analysis of ex vivo-expanded NK and NK-like T cells administered to cancer patients: a phase I clinical study. Immunotherapy. 2009;1(5):753-764. 10.2217/imt.09.47 20636021

[105] Noronha NC , Mizukami A, Caliári-Oliveira C, et al. Priming approaches to improve the efficacy of mesenchymal stromal cell-based therapies. Stem Cell Res Ther. 2019;10(1):131. 10.1186/s13287-019-1224-y 31046833 PMC6498654

[106] Chinnadurai R , Copland IB, Garcia MA, et al. Cryopreserved mesenchymal stromal cells are susceptible to T-cell mediated apoptosis which is partly rescued by IFNγ licensing. Stem Cells. 2016;34(9):2429-2442. 10.1002/stem.2415 27299362 PMC5016228

[107] Duijvestein M , Wildenberg ME, Welling MM, et al. Pretreatment with interferon-γ enhances the therapeutic activity of mesenchymal stromal cells in animal models of colitis. Stem Cells. 2011;29(10):1549-1558. 10.1002/stem.698 21898680

[108] François M , Copland IB, Yuan S, et al. Cryopreserved mesenchymal stromal cells display impaired immunosuppressive properties as a result of heat-shock response and impaired interferon-γ licensing. Cytotherapy. 2012;14(2):147-152. 10.3109/14653249.2011.623691 22029655 PMC3279133

[109] Moll G , Alm JJ, Davies LC, et al. Do cryopreserved mesenchymal stromal cells display impaired immunomodulatory and therapeutic properties? Stem Cells. 2014;32(9):2430-2442. 10.1002/stem.1729 24805247 PMC4381870

[110] Hoogduijn MJ , de Witte SFH, Luk F, et al. Effects of freeze-thawing and intravenous infusion on mesenchymal stromal cell gene expression. Stem Cells Dev. 2016;25(8):586-597. 10.1089/scd.2015.0329 26914168

[111] Sadeghi B , Witkamp M, Schefberger D, Arbman A, Ringdén O. Immunomodulation by placenta-derived decidua stromal cells role of histocompatibility, accessory cells and freeze-thawing. Cytotherapy. 2023;25(1):68-75. 10.1016/j.jcyt.2022.10.004 36333233

[112] Ringden O , Uzunel M, Sundberg B, et al. Tissue repair using allogeneic mesenchymal stem cells for hemorrhagic cystitis, pneumomediastinum and perforated colon. Leukemia. 2007;21(11):2271-2276. 10.1038/sj.leu.2404833 17611560

[113] Wang R , Yao Q, Chen W, et al. Stem cell therapy for Crohn’s disease: systematic review and meta-analysis of preclinical and clinical studies. Stem Cell Res Ther. 2021;12(1):463. 10.1186/s13287-021-02533-0 34407875 PMC8375136

[114] Ringdén O , Moll G, Gustafsson B, Sadeghi B. Mesenchymal stromal cells for enhancing hematopoietic engraftment and treatment of graft-versus-host disease, hemorrhages and acute respiratory distress syndrome. Front Immunol. 2022;13:839844. 10.3389/fimmu.2022.839844 35371003 PMC8973075

[115] Sadeghi B , Roshandel E, Pirsalehi A, et al. Conquering the cytokine storm in COVID-19-induced ARDS using placenta-derived decidua stromal cells. J Cell Mol Med. 2021;25(22):10554-10564. 10.1111/jcmm.16986 34632708 PMC8581334

[116] Aronsson-Kurttila W , Baygan A, Moretti G, et al. Placenta-derived decidua stromal cells for hemorrhagic cystitis after stem cell transplantation. Acta Haematol. 2018;139(2):106-114. 10.1159/000485735 29408819

[117] Sadeghi B , Ersmark B, Moretti G, Mattsson J, Ringdén O. Treatment of radiculomyelopathy in two patients with placenta-derived decidua stromal cells. Int J Hematol. 2020;111(4):591-594. https://doi.org/10.1007/s12185-019-02804-w 31853810 10.1007/s12185-019-02804-w PMC7102257

